# Bromocriptine: does this drug of Parkinson’s disease have a role in managing cardiovascular diseases?

**DOI:** 10.1097/MS9.0000000000001642

**Published:** 2023-12-19

**Authors:** Lakshmi Venkata Simhachalam Kutikuppala, Sushil Sharma, Madhavrao Chavan, Gaurav Rangari, Arup Kumar Misra, Sai Ram Innamuri, Tejus Vijayakumar, Golla Varshitha

**Affiliations:** a Department of Pharamacology, All India Institute of Medical Sciences (AIIMS), Mangalagiri, Andhra Pradesh, India; b Department of General Medicine, International School of Medicine (ISM), Bishkek, Kyrgyzstan

**Keywords:** Bromocriptine, cardiovascular disease (CVD), Dopamine antagonist

## Abstract

Cardiovascular disease (CVD) is the most common cause of morbidity and mortality worldwide. Bromocriptine is a partial antagonist for D1 dopamine receptors while also serving as a selective agonist on D2 dopamine receptors as a dopamine receptor agonist. Apart from prolactin inhibiting action, bromocriptine has some beneficial effects on the blood pressure, plasma norepinephrine levels and vascular resistance. Dopamine D2 receptor activation of bromocriptine is associated with the antihypertensive effect, which lowers blood pressure via inhibiting sympathetic nerve activity and Na/K ATPase activity. Plasma levels of the pro-inflammatory cytokines such as interleukin (IL)-1B and IL-18, chemokine CCL2/ MCP-1/, and the pro-inflammatory hormone prolactin, all of which are elevated and linked to accelerated cardiometabolic illness, were decreased because of bromocriptine therapy. The most common side effects of Bromocriptine use are dizziness, nausea, headache, vomiting and hypotension. Bromocriptine is mainly contraindicated in patients with syncope with hypotension, psychosis, and type I diabetes mellitus. The authors suggest that developing therapies directed to increase D2 receptor expression and function by drugs like Bromocriptine can provide practical and novelistic approaches to prevent and manage myocardial and renal injury in the cardiovascular disease patients.

## Introduction

Highlights

- Apart from prolactin inhibiting action, bromocriptine has some beneficial effects on the blood pressure, plasma norepinephrine levels and vascular resistance.
- Bromocriptine can be a key player in bringing up better outcomes among the cardiovascular disease patients through adequate control of cardiac parameters and reducing the risk of disease.
- Dopamine D2 receptor activation of bromocriptine lowers blood pressure via inhibiting sympathetic nerve activity and Na/K ATPase activity.
- Plasma levels of the pro-inflammatory cytokines such as IL-1B and IL-18, chemokine CCL2/ MCP-1/, and the pro-inflammatory hormone prolactin were decreased because of bromocriptine therapy.

The most common cause of mortality and morbidity worldwide is the cardiovascular disease (CVD)^1^. One of the earliest and most prevalent cardiovascular illnesses is high blood pressure, which is followed by left ventricular hypertrophy (LVH) brought on by the volume overload^1,2^. Bromocriptine is a partial antagonist for D_1_ dopamine receptors while also serving as a selective agonist on D_2_ dopamine receptors as a dopamine receptor agonist. Bromocriptine is commonly used to treat type 2 diabetes, ovarian hyperstimulation syndrome, hyperprolactinemia, and acromegaly^3^. It has been thought that Bromocriptine also has an impact on cardiac remodelling although the accurate mechanism of action is uncertain^4^. Bromocriptine is thought to have a cardioprotective effect by lowering the sympathetic activity of heart, which may lower the likelihood of potentially fatal ventricular arrhythmias^4,5^. Numerous clinical disorders such as hypertension, obesity, insulin resistance, and chronic kidney disease (CKD), have been related to sympathetic overactivity, high norepinephrine (NE) plasma levels and hyperprolactinemia which suggests a diminished dopaminergic tone^5^. Additionally, it has been suggested that a reduction in dopaminergic tone and a rise in sympathetic activity may be the cause of the link between cardiac dysregulation and the subsequent development of hypertension and metabolic syndrome^5,6^. Hence, it is likely that patients could benefit from the use of dopamine agonists in these circumstances, and this is backed by numerous studies showing positive effects of bromocriptine on cardiovascular and metabolic parameters^6^. This review is sought to determine the impact of bromocriptine and the underlying mechanisms involved in regulating the cardiovascular system. We reviewed the databases namely PubMed, ScienceDirect, DOAJ, Embase, and Web of Science for the literature on the topic with key words like Bromocriptine, Cardiovascular disease, Dopamine antagonist, Hypertension, and Ventricular hypertrophy.

### Various mechanism underlying the action of bromocriptine on cardiovascular system

**Figure 1:**
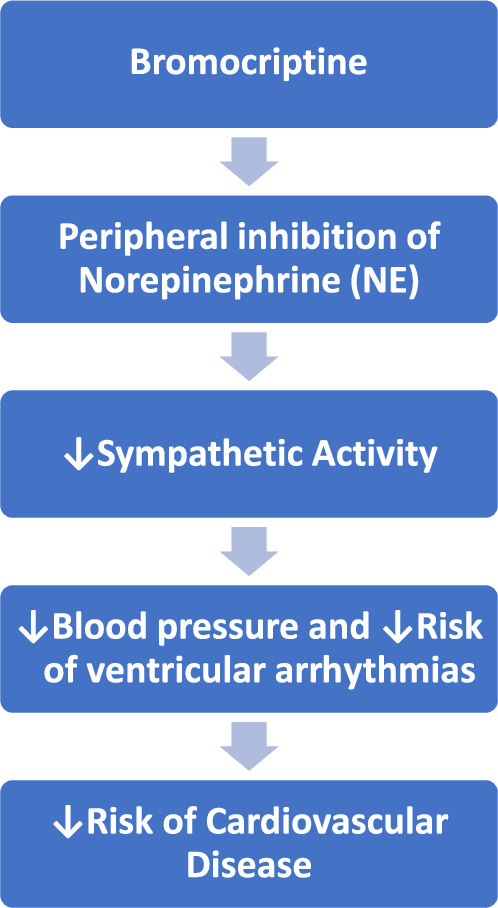
Mechanism underlying the action of Bromocriptine in reducing cardiovascular disease risk.

Apart from prolactin inhibiting action, bromocriptine has some beneficial effects on the blood pressure, plasma norepinephrine levels and vascular resistance. Dopamine D_2_ receptor activation of bromocriptine is associated with the antihypertensive effect, which lowers blood pressure via inhibiting sympathetic nerve activity and Na/K ATPase activity^7^. Additionally, bromocriptine has been demonstrated to lower LV filling pressure and enhance stroke volume index^8^. The pathophysiology of cardiovascular disease, including cardiac arrhythmias and congestive heart failure, has been linked to autonomic nervous system dysfunction. Sympathetic hyper activation is one of the key factors that increase the risk of ventricular arrhythmias (VA) and result in sudden cardiac death among the people living with myocardial infarction (MI) and heart failure^9^. Since ventricular arrhythmias are linked to elevated the sympathetic activity, the effect of bromocriptine may indeed be clinically significant. The sympathoinhibitory effect of bromocriptine is exclusively due to the peripheral inhibition of NE release. Bromocriptine has been found to increase the threshold of the sympathetic system towards ventricular arrhythmias in the experimental animals^10^. Bromocriptine has been observed to reduce the blood pressures in the patients with essential hypertension. (Fig. 1) Furthermore, a reduction in the dopaminergic sympathoinhibitory mechanisms can be a key factor in the pathophysiology and regulation of essential hypertension. Bromocriptine can have a direct vasodilator effect through a possible vascular dopaminergic receptor stimulation^11^.

### Role of bromocriptine in managing hypertension in CVD patients

**Figure 2:**
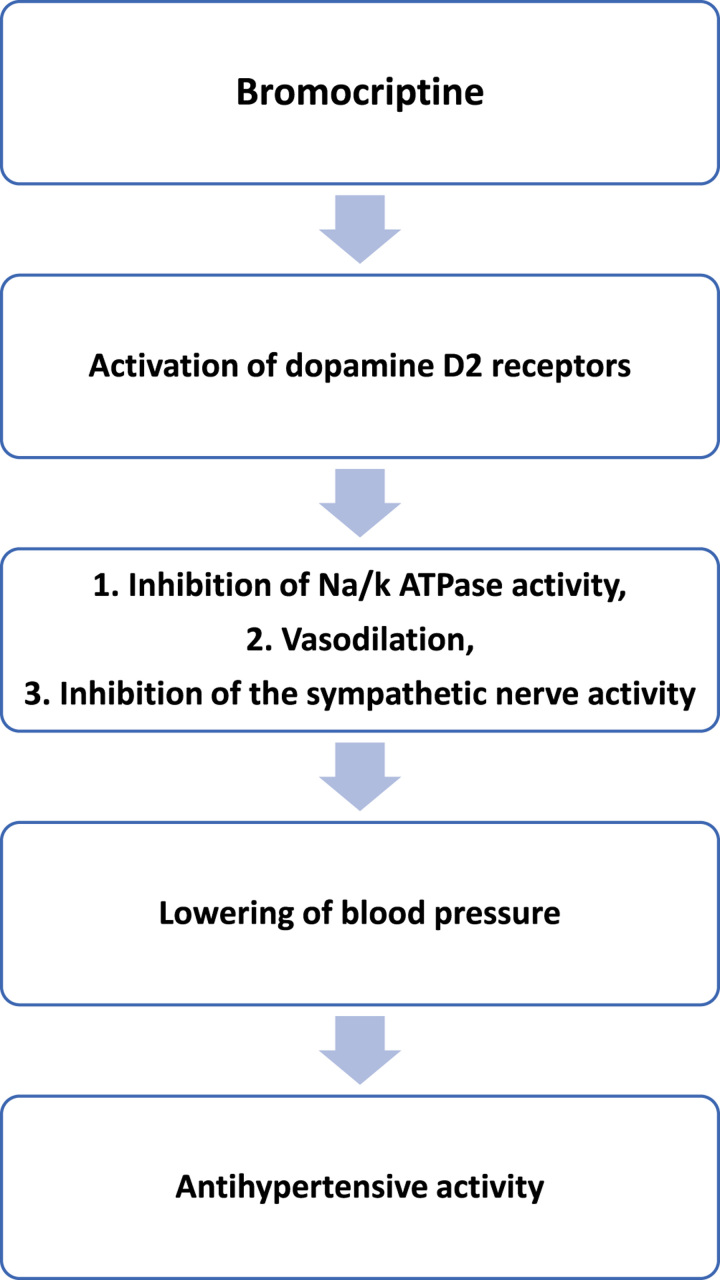
Mechanism of Bromocriptine in managing hypertension.

A decrease in blood pressure and reduction in the cardiac sympathetic activity were linked to the administration of bromocriptine, which is likely connected to the sympathetic efferent loop of the baroreflex. The plasma levels of norepinephrine were nevertheless decreased^12^. In this regard, it is important to note that despite a decrease in blood pressure, bromocriptine treatment had no effect on the RR interval. These results are probably explained by the combined impact of the opposing in vivo effects of bromocriptine^7^. In fact, bromocriptine can drop norepinephrine levels, which can lead to hypotension; however, low blood pressure values can also trigger a reflex activation of the sympathetic output^13^. The mechanism of Bromocriptine in managing hypertension is shown in Fig. 2.

### Use of bromocriptine peripartum cardiomyopathy (PPCM)

A potentially debilitating cardiomyopathy known as peripartum cardiomyopathy (PPCM) affects women during the puerperal stage, which is significantly triggered by a portion of prolactin (16-kDa)^14^. PPCM is marked by the acute onset of heart failure between one month to five months post-delivery in the previously healthy women. Within 6 months following delivery, only 23–54% of patients demonstrate a return of normal heart function^15^. Evaluation of interleukin (IL)-6, tumour necrosis factor (TNF)-Alpha, C-reactive protein (CRP) and Fas-Apo-1 levels in the plasma of these PPCM patients who underwent investigation revealed that a pro-inflammatory response resulting from the disease^16^. Various studies have validated that adding bromocriptine to conventional heart failure treatment seems to enhance left ventricular ejection fraction and a composite clinical outcome among women with acute severe PPCM^17^. None of the studies reported recurrence of left ventricular dysfunction in the subsequent pregnancies of women with PPCM for the first time treated with regular HF treatment combined with bromocriptine when compared to those treated with only HF treatment^18^.

### Cardioprotective role of bromocriptine

Potential biological pathways that may contribute to the cardioprotective benefits of bromocriptine were provided by the findings of a recent study by Chamarthi *et al.* ^21^. This study validated that circadian-timed bromocriptine QR (quick release) can cause simultaneous reduction in sympathetic tone and an immunological and systemic pro-inflammatory/pro-oxidative state. Plasma levels of the pro-inflammatory cytokines such as IL-1B and IL-18, chemokine CCL2/ MCP-1/, and the pro-inflammatory hormone prolactin, all of which are elevated and linked to accelerated cardiometabolic illness, were decreased as a result of bromocriptine therapy^7,22,23^. The key risk factors for CVD including the inflammation, sympathetic tone, endothelial nitric oxide synthase uncoupling, and increased postprandial lipids are decreased by circadian-timed bromocriptine medication^24^. A study by Fouad *et al.* showed that BROMOCRIPTINE have played a significant role in reducing the levels of cardiac markers like Troponin I, TNF alpha and LDH-1^25^. The cardioprotective effects of bromocriptine observed by various studies are shown in Table 1.

**Table 1:** Showing cardioprotective effects of bromocriptine

References	Observed cardioprotective effects of Bromocriptine
Cincotta *et al.* ^7^	• Plasma biomarkers of oxidative stress and inflammation are decreased • Sympatholytic effects lower norepinephrine levels in the blood
Chamarthi *et al.* ^13^	• Reduction in plasma levels of the pro-inflammatory cytokines IL-1B and IL-18, chemokine MCP-1/CCL2, and pro-inflammatory hormone prolactin
Badianyama^18^	• Suppression of prolactin levels, and promotion of cardiac recovery at 3-month and 6-month follow-up in mothers with Peripartum Cardiomyopathy
Alatrach *et al.* ^23^	• Significant reduction in blood pressure and in pulse pressure
Siamashvili *et al.* ^24^	• Improved vascular indices and reduction in cardiovascular risk
Fouad Shalaby *et al.* ^25^	• Decrease in secretion of myocardial biomarkers like Troponin I, LDH-1, and TNF alpha 1

IL, interleukin; TNF, tumour necrosis factor.

### Side effects of bromocriptine

The most common side effects of Bromocriptine use are dizziness, nausea, headache, vomiting and hypotension. The unusual side effects can be fibrosis (retroperitoneal, cardiac valve or pleural) and psychosis. Bromocriptine is mainly contraindicated in patients with syncope with hypotension, psychosis, and type I diabetes mellitus^3^.

### Pharmacodynamics of bromocriptine in CVD patients

Most of the patients with cardiovascular disease can tolerate the bromocriptine well^19^. None of the significant adverse events, like the episodes of thromboembolism are associated with the use of Bromocriptine in the management of PPCM^20^. A study by Budayr *et al.* ^26^ observed that when compared to cabergoline, bromocriptine use was linked to a lesser prevalence of mild valvular regurgitation in community-based people receiving treatment for hyperprolactinemia. Bromocriptine can prevent chronic nephropathy and protect the kidney from ischaemia-reperfusion (I/R) injury by activating the p44/42 mitogen-initiated protein kinase. The American Diabetes Association states that 4.8 mg of bromocriptine is the highest authorized daily dose for humans. Overdosing of bromocriptine (>10 mg/day) can lead to damage of myocardial tissue and cause valvular disease of heart^25,27^.

Although the studies hypothesize and indicate the consideration of bromocriptine in the management of CVD, its role cannot be generalized to other organ specific sympathetic neural responses with the limited literature available. Hence, further extensive research is required to validate and strengthen the role of bromocriptine in managing CVDs.

## Conclusion

Bromocriptine is thought to have a cardioprotective effect because its tendency to lower heart sympathetic activity and peripheral norepinephrine release. We suggest that developing therapies directed to increase D2 receptor expression and function by drugs like Bromocriptine can provide practical and novelistic approaches to prevent and manage myocardial and renal injury in the cardiovascular disease patients. As discussed in this review, Bromocriptine can be a key consideration in bringing up better outcomes among the CVD patients through adequate control of cardiac parameters and reducing the risk of disease.

## Ethical approval

None.

## Consent

Informed consent is not required for this review.

## Sources of funding

None.

## Author contribution

L.V.S.K.: study concept, drafting and editing the paper. S.S.: drafting and editing the paper. M.C.: drafting and editing the paper. G.M.R.: drafting and editing the paper. A.K.M.: drafting and editing the paper. S.R.I.: drafting and editing the paper. T.V.: drafting and editing the paper. V.G.: drafting, editing and submitting the paper.

## Conflicts of interest disclosure

There are no conflicts of interest.

## Research registration unique identifying number (UIN)

None.

## Guarantor

Varshitha Golla.

## Data availability statement

None.

## Provenance and peer review

Not commissioned, externally peer-reviewed
